# A study on brain waves with electroencephalogram through the appreciation of virtual reality K-pop: a revised analysis with multiple comparison corrections

**DOI:** 10.3389/fphys.2026.1808008

**Published:** 2026-05-08

**Authors:** Taeseung Park, Hyunsoo Kim, Deok Su Yoo

**Affiliations:** 1 Department of Global Taekwondo, JEI University, Incheon, Republic of Korea; 2 Graduate School of Physical Education, Kyung Hee University, Seoul, Republic of Korea; 3 Department of Taekwondo, College of Physical Education, Kyung Hee University, Seoul, Republic of Korea

**Keywords:** brain waves, EEG analysis, effect size, K-pop, media richness theory, metaverse content, multiple comparison correction, virtual reality

## Abstract

**Background:**

Korean popular culture (K-pop) has become increasingly popular worldwide and the convergence of K-pop and virtual reality (VR) has contributed to the popularity of metaverse technologies. Previous studies on K-pop and VR were largely conducted in the fields of cognitive psychology and qualitative research. Recently, the use of functional magnetic resonance imaging (MRI), electroencephalogram (EEG), and eye-trackers have gained attention.

**Methods:**

In this study, EEG analysis was used to evaluate alpha waves, beta waves, and theta/beta ratios of 20 participants who had experienced VR or normal K-pop music video content, and the data analyzed based on the Media Richness Theory. Statistical analyses employed paired t-tests with False Discovery Rate (FDR) correction for multiple comparisons using the Benjamini-Hochberg procedure, and effect sizes (Cohen’s d) were calculated to assess practical significance.

**Results:**

The results revealed differences in alpha wave (*p* <.05), beta wave (*p* <.05), and theta/beta ratios between the general and VR music video groups. After FDR correction, 11 electrodes showed significant alpha wave differences (d = 0.54-1.51), 13 electrodes showed significant beta wave differences (d = 0.50-1.04), and 8 electrodes showed significant theta/beta ratio differences (d = 0.63-0.87). Alpha waves were higher in the normal music video group, whereas beta waves and the theta/beta ratio were higher in the VR music video group.

**Conclusion:**

The EEG results suggest that VR K-pop music videos are rich in media content, providing more information than normal K-pop music videos. The robustness of findings was confirmed through rigorous statistical correction, with medium to large effect sizes indicating substantial practical significance beyond statistical significance. This approach has the potential to develop a strategy for metaverse K-pop content.

## 1 Introduction

The diverse content of Korean popular culture (K-pop) contributes to its expanding influence in Europe, Russia, the United States, South America, and Southeast Asia. This phenomenon goes beyond the simple export of K-popular to other countries with K-pop exerting a positive effect on the marketing competitiveness and image of Korea. K-pop does not imitate Western culture, and each artist or group’s individuality is recognized and regarded as a cultural asset with global marketing power. K-pop is therefore a symbol of the Korean cultural industry, with an impact on popular culture worldwide (Kim, 2022; Suh, 2022).

Initially, BTS boldly implemented an online personal relations strategy to differentiate themselves from large record labels in Korea, thereby gaining global fandom. BTS’s musicality, choreography, and online communication with fans promoted the quantitative and qualitative growth of their fandom (Lee et al., 2021).

The success of K-pop stems from production of themed content that considers fans around the world, as well as discovery and nurturing of suitable artists at an early stage, and the Born Global Mix of Talent Development and Acquisition strategy that supports production work, including music composition and choreography (Limb, 2013). These strategies enabled successful global fandom for K-pop artists.

To increase the global influence of K-pop, various forms of metaverse performance are provided through fusion with virtual reality (VR), augmented reality, holograms, and computer-graphic technologies. A metaverse platform with content using VR and augmented reality technologies expands and strengthens fandom by providing rich experiences that stimulate various senses in addition to visual information (McLellan, 1996; Kim, 2018). In particular, combining VR technology with K-pop video content enhances perspective, three-dimensional effects, and reality compared with normal K-pop video content, and allows fans to experience spectacular visual effects and novel fun (Kang et al., 2021). VR K-pop video content maximizes sensory input, providing a realistic experience, allowing consumers to experience interactivity with VR K-pop video content, thereby deepening immersion. The positive evaluation of VR K-pop video content can be explained by the Media Richness Theory, which refers to the ability of media to convey a large amount of information through a variety of cues, with rich media having a high ability to deliver to users, whereas lean media does not (Daft and Lengel, 1986). The Media Richness Theory therefore describes the experience of deep immersion, pleasure, or satisfaction, and positive or negative attitudes based on consumers’ interaction patterns with various media. How Media Richness Theory applies to content in new areas such as VR K-pop video content needs to be verified (Choi et al., 2022).

In a study by Berns and Moore (2012), brain activity in the orbitofrontal cortex and ventral striatum was measured while subjects listened to music from a previously unencountered era. The results revealed a correlation between music sales volume and reward-related brain activity, suggesting that the success potential of popular music can be predicted by analyzing brain activity. In addition, Telpaz et al. (2015) reported differences between EEGs when consumers evaluated products they preferred compared with products they dislike in areas other than music, suggesting a correlation between brain waves and the purchasing decision process of consumers.

Research predicting the success potential of popular music and various consumer goods through EEG analysis is actively being conducted. However, most previous studies on how K-pop and fans influence each other have been conducted in the fields of cognitive psychology and qualitative research, including in-depth case study analyses, surveys, and literature research (Kraus et al., 2021; Kim M. J. et al., 2018; Kim Y. S. et al., 2018), with limited meaningful research on the influence of VR K-pop content on consumer perceptions and purchase intentions. Research using functional magnetic resonance imaging (MRI), electroencephalogram (EEG), and eye-trackers have gained attention. EEG analysis measures the electrical activity of neurons in the cerebral cortex, amplifies the signal, and analyzes the data noninvasively through equipment that records activity on the scalp. EEG analyzers are widely used because they are portable, easy to operate, and inexpensive compared with other devices (Hsu et al., 2018). The unconscious psychological state of fans experiencing normal and VR K-pop music video content may therefore be compared by evaluating EEG changes of the participants.

While previous neuroscience studies on media processing have provided valuable insights, a critical methodological limitation in the field is the frequent failure to address the multiple comparison problem when conducting numerous statistical tests across multiple brain regions (Ohmea et al., 2010; Koelstra et al., 2010). When multiple statistical tests are performed (e.g., comparing EEG activity across 17 electrodes and 3 frequency bands), the probability of false positive findings increases substantially without appropriate correction. The False Discovery Rate (FDR) method proposed by Benjamini and Hochberg (1995) offers a powerful solution that maintains adequate statistical power while controlling the rate of false discoveries, making it particularly suitable for exploratory neuroscience research.

This study aimed to measure the brain waves of participants experiencing VR and normal K-pop music video content and perform in-depth analysis based on the Media Richness Theory. Importantly, this study addresses the multiple comparison problem by applying rigorous FDR correction to all statistical tests and reports effect sizes (Cohen’s d) to assess the practical significance of findings, thereby contributing methodologically sound evidence to the emerging field of VR entertainment neuroscience.

The human brain participates in higher cognitive functions and control, as well as integrating emotions and determining subjective values. Previous studies have predicted human emotions by analyzing brain waves of people watching advertisements, and confirmed left hemispheric dominance during emotional cognitive processing (Ohmea et al., 2010). In addition, a study that measured the brain waves of subjects while watching music videos found that the frequency power in various cortical regions strongly correlated with music video preference (Koelstra et al., 2010). The present study therefore aimed to empirically verify the effect of VR K-pop music videos on consumer concentration and immersion based on the Media Richness Theory. Alpha waves, beta waves, and theta/beta ratios were measured as indicators of significant changes in the areas associated with concentration and immersion.

Among the EEG frequency bands examined, alpha waves are known to respond very sensitively to external visual stimuli compared with other waveforms. Red’ka and Mayorov (2015) and Rothschild and Hyun (1990) observed alpha blocking, in which alpha waves decrease as the brain concentrates on external stimuli, suggesting that the degree of visual stimulation affects alpha wave intensity. Given that VR K-pop content provides enhanced visual stimulation compared to normal content, it was hypothesized that there would be a difference in the power spectral density (PSD) of alpha waves between participants who experienced VR K-pop video content and those who experienced normal K-pop video content.

Regarding beta wave activity, Wolfram and Richard (2008) reported that beta waves are related to the degree and state of arousal to external stimuli, and that an increase in beta waves can identify tension and excitement states. Based on this evidence that visual stimulation affects beta wave activity, it was further hypothesized that VR K-pop video content would elicit different PSD values of beta waves compared to normal K-pop video content.

Additionally, the Theta/Beta Ratio (TBR), which refers to the ratio of the Sensory Motor Rhythm (SMR) wave to the Theta wave and the Mid-beta wave, was examined as an indicator of attentional engagement. Lubar (1991) found that the theta/beta ratio is an index that can objectively confirm the attention span and concentration state of participants, and that visual stimulation can change the degree of attention span and concentration. Based on these previous studies, it was hypothesized that general K-pop music videos and VR K-pop music videos would show a difference in the theta/beta ratio, which indicates the participants’ attention span and concentration.

## 2 Materials and methods

This study was conducted with the approval of the Research Ethics Committee from SeJong University (IRB-SUIRB-HR-2022-004).

### 2.1 Participants

Participants in this study were college students in their 20s. Eligibility was limited to individuals with no current or past brain-related disease or mental disorder, no claustrophobia or insomnia, no need to wear glasses while using the VR equipment, no current medication use, and normal blood pressure. Exclusion criteria were prespecified to reduce potential confounding of EEG outcomes, including current use of medications or substances that could plausibly affect central nervous system activity (e.g., sedating cold medications, psychoactive drugs, or hormonal treatments). Following the recruitment announcement, 20 males and 3 females expressed interest; however, the three female volunteers were excluded at screening because their medication use met the exclusion criteria. Accordingly, this is a notable limitation of the study, and the present findings should be interpreted as primarily applicable to healthy young adult males. Based on prior human electrophysiology studies using similar conditions and experimental procedures, the target sample size was selected to align with the reported range of 7 to 26 participants per group (Larson and Carbine, 2017).

Therefore, twenty male participants were voluntarily recruited, and the average age of the participants was 22.35 years old (SD = 2.01), height was 176.43 cm (SD = 3.09), and weight was 75.25 kg (SD = 3.01).

### 2.2 Research procedure and visual stimuli

#### 2.2.1 Research procedure

On 18 May 2022, the physical condition of the participants was checked to prevent all possible risks during the experiment. The study was conducted with the participants sitting in a comfortable chair and auxiliary personnel were always on standby in case the participants felt dizziness or nausea during the content experience. In case the participants lost consciousness when experiencing the content, the nearest hospital was contacted in advance so that they could be transferred as soon as possible. In the middle of the study, if the participants felt uncomfortable while conducting the experiment or had a problem with stability, the experiment was immediately stopped, and measures were taken to prevent physical and mental injury to the participants. The contents related to these safety measures were notified to the participants in advance before the experiment.

After the experiment was explained, background EEG was measured for 120 s after an absence of mixing artifacts for more than 10 s. The non-stimulation state was maintained for 180 s, and set as a reference state before stimulation. Participants subsequently experienced the normal version of the Next Level music video by Aespa for 120 s wearing Oculus equipment, and index values, including brain-wave PSD, were extracted. After the measurement, the non-stimulated state was maintained for 180 s for EEG recovery to the reference state. Participants subsequently experienced the VR version of the Next Level music video by Aespa for 120 s wearing Oculus equipment, and the index value of the EEG PSD was extracted.

Content exposure time was designed with equal exposure for 120 s, starting from 60 s out of the 4 min 3 s full-length music video. All processes in this study were video recorded using a Samsung Galaxy S22 camera installed on a Bellbone M45 tripod facing a direction preventing recognition of the participant.

#### 2.2.2 Visual stimulus

**Figure 1:**
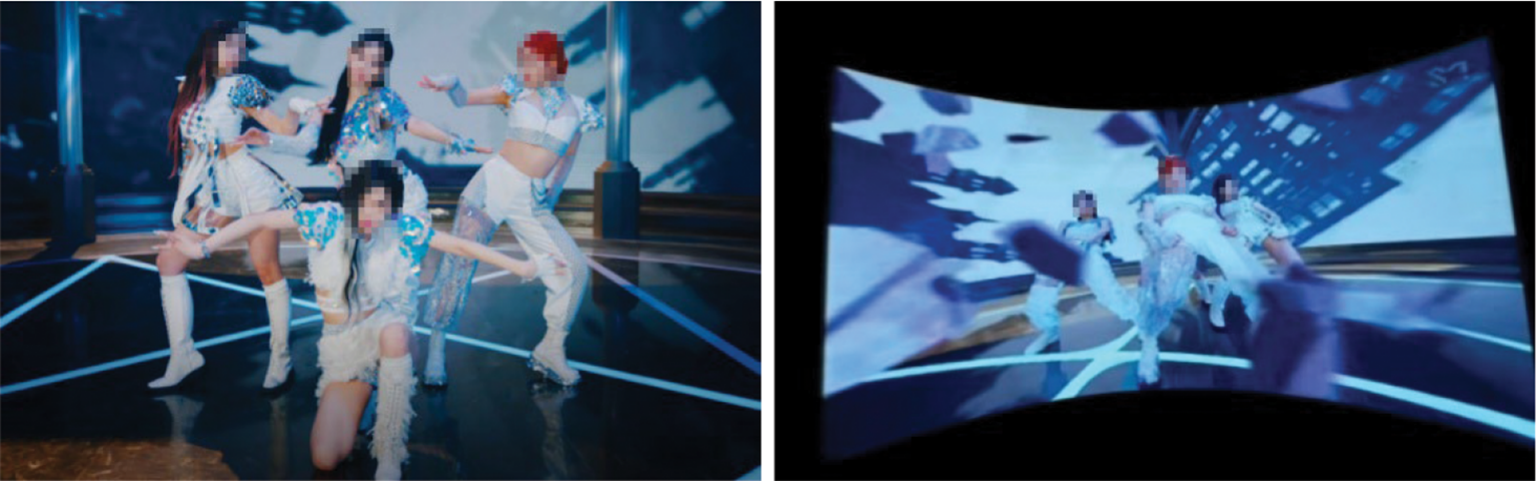
Next Level music video image (left, normal content; right, VR content).

Visual stimuli included the normal and VR versions of the Next Level music video by Aespa of SM Entertainment in Korea. This music video was selected based on content validity verification by a broadcast content production expert, a VR content expert, and two doctors majoring in mass media. The top three YouTube views of VR K-pop music videos were evaluated based on content popularity and content quality that enhances immersion in the virtual world, receiving a high evaluation compared with other K-pop VR content. **Figure 1** shows images of the normal and VR Next Level music videos (**Figure 1**).

### 2.3 EEG measurement

**Figure 2:**
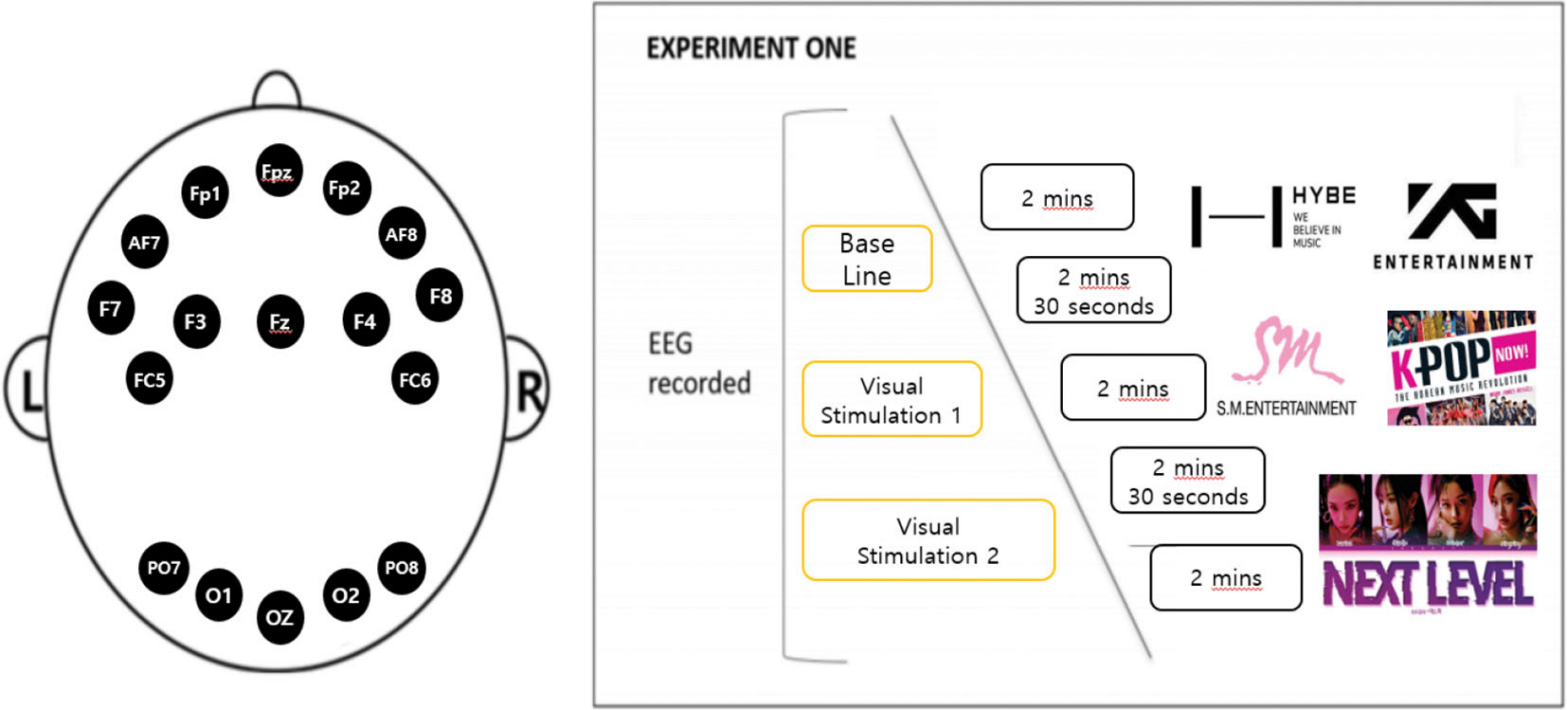
EEG electrode placement locations using the International 10-20 system (left) and experiment flow chart (right).

EEG measurements were performed using Quick-32r equipment (CGX, 2022), a device that wirelessly measures human brain waves, and can therefore be used in various situations, such as during exercise, sleeping, and eating. EEG measurements were performed according to the 10–20 International Electrode Placement Method (Jasper, 1958), and AF7, Fpz, F7, Fz, FC6, Fp1, F4, Oz, PO8, O2, O1, PO7, Fp2, F3, F8, FC5, and AF8 were evaluated (**Figure 2**). A reference electrode was also attached. The sampling frequency was 500 Hz, and the measured analog signal was converted into a 24-bit digital signal.

### 2.4 EEG data analysis

EEG power spectrum-based indicators were analyzed using BioScan (BioBrain Inc, 2016). To remove electrical noise, a 60 Hz notch filter was applied and a 0.5–50 Hz band-pass filter was applied. For each 120-s viewing condition, spectral analyses were conducted on a 90-s steady-state segment defined *a priori* as the middle portion of the exposure; the first 15 s and the last 15 s were excluded to reduce onset/offset transients and potential movement-related artifacts associated with content initiation and termination. The analysis index calculated according to each condition was mapped using the cloud-based BioScan-Map (BioBrain Inc, 2016).

Paired sample t-tests were performed for the analyzed EEG indices using the SPSS ver.23 program. Given that multiple comparisons were conducted across 17 electrodes and three EEG measures (alpha power, beta power, and theta/beta ratio), correction for multiple testing was essential to reduce the risk of false positive findings. Accordingly, a total of 51 comparisons (17 × 3) were considered for multiplicity control.

False Discovery Rate (FDR) correction using the Benjamini-Hochberg procedure (Benjamini and Hochberg, 1995) was applied to adjust p-values for multiple comparisons. The FDR method controls the expected proportion of false discoveries among rejected null hypotheses, offering greater statistical power than traditional Bonferroni correction while maintaining rigorous control over Type I errors. A q-value threshold of 0.05 was used as the criterion for statistical significance after FDR adjustment. Additionally, As a conservative sensitivity analysis, Bonferroni correction was also considered using the same total number of comparisons (adjusted alpha = 0.05/51 = 0.00098). 

Effect sizes were calculated using Cohen’s d for paired samples, defined as d = M_diff/SD_diff, where M_diff is the mean of the paired differences and SD_diff is the standard deviation of the differences. This within-subjects effect size metric appropriately accounts for the paired nature of the data and provides a standardized measure of the magnitude of differences independent of sample size. Effect sizes were interpreted according to conventional benchmarks: small (d = 0.2), medium (d = 0.5), and large (d = 0.8) (Cohen, 1988).

The statistical significance level was set to alpha = .05 for uncorrected tests. However, FDR-corrected q-values < 0.05 served as the primary criterion for determining statistical significance in all reported results, ensuring that findings are robust against the inflation of Type I error rates associated with multiple testing.

## 3 Results

A study was conducted to verify the hypotheses, and it was confirmed that there was a difference in the alpha wave, beta wave, and theta/beta ratio when watching the normal K-pop video content and when watching the VR K-pop video content. All results presented below were subjected to False Discovery Rate (FDR) correction for multiple comparisons using the Benjamini-Hochberg procedure, ensuring robust control of false positive findings. Effect sizes (Cohen’s d) were calculated for all comparisons to assess practical significance.

### 3.1 EEG analysis results between two groups in alpha wave

As a result of analyzing the difference in alpha waves between the two groups, there was a significant difference between normal K-pop contents (NX group) and VR K-pop contents (VR group) in specific brain regions. After FDR correction (q < 0.05), 11 out of 17 electrodes maintained statistical significance. The specific brain regions include a total of 11, including AF7, Fpz, F7, Fz, Fp1, F4, PO7, Fp2, F3, F8, and AF8, as shown in **Table 1**.

**Table 1:** EEG analysis results between two groups in alpha wave.

Electrode	Normal (NX) M (SD)	VR M (SD)	t-statistic	p-value	p-FDR	Cohen's d
AF7	.174 (.017)	.153 (.017)	3.781***	.001	.004	0.85
Fpz	.178 (.015)	.154 (.010)	6.769***	<.001	<.001	1.51
F7	.177 (.026)	.164 (.016)	2.600*	.018	.030	0.58
Fz	.191 (.021)	.171 (.014)	3.782***	.001	.004	0.85
Fp1	.175 (.018)	.152 (.015)	5.586***	<.001	<.001	1.25
F4	.192 (.025)	.175 (.008)	3.116**	.006	.011	0.70
PO7	.169 (.017)	.159 (.012)	2.429*	.025	.039	0.54
Fp2	.177 (.014)	.156 (.013)	4.747***	<.001	.001	1.06
F3	.191 (.025)	.171 (.012)	3.294**	.004	.008	0.74
F8	.180 (.025)	.165 (.016)	3.597**	.002	.005	0.80
AF8	.178 (.019)	.161 (.012)	3.436**	.003	.007	0.77

*p<.05, **p<.01, ***p<.001 (after FDR correction); NX, Next Level music video audience; VR, VR Next Level music video audience.

**Figure 3:**
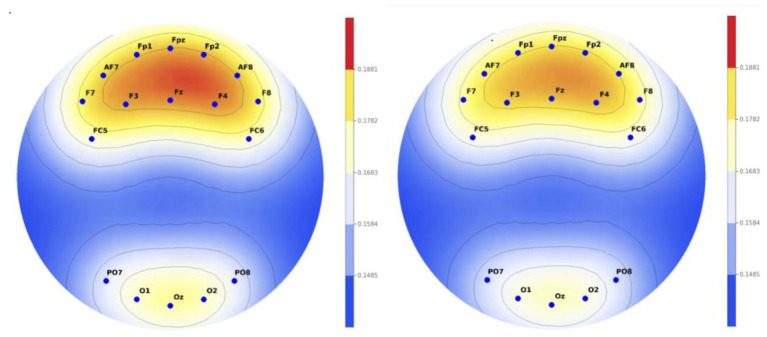
Comparison of EEG activity between two groups in alpha wave (left, normal content; right, VR content).

As a result of the analysis, there was a difference in alpha wave between the group who watched normal music video and the group who watched the VR music video. It was confirmed that the group that watched the normal music video had a higher alpha wave than the group that watched the VR music video. All 11 electrodes demonstrated medium to large effect sizes (Cohen’s d = 0.54-1.51), indicating not only statistical significance but also substantial practical significance. The largest effects were observed in prefrontal regions (Fpz: d = 1.51, Fp1: d = 1.25, Fp2: d = 1.06), suggesting particularly strong differential processing of VR versus normal content in executive control and attention networks (**Figure 3**).

### 3.2 EEG analysis results between two groups in the beta wave

As a result of analyzing the difference in beta waves between the two groups, there was a significant difference between general K-pop contents (NX group) and VR K-pop contents (VR group) in specific brain regions. After FDR correction (q < 0.05), 13 out of 17 electrodes maintained statistical significance. The specific brain regions are 13 regions, including AF7, Fpz, F7, Fz, Fp1, F4, PO8, O2, PO7, Fp2, F3, FC5, and AF8, as shown in **Table 2**.

**Table 2:** EEG analysis results between two groups in the beta wave.

Electrode	Normal (NX) M (SD)	VR M (SD)	t-statistic	p-value	p-FDR	Cohen's d
AF7	0.393 (0.036)	0.424 (0.033)	-3.760	.001	.005	-0.84
Fpz	0.376 (0.036)	0.412 (0.030)	-3.985	.001	.005	-0.89
F7	0.383 (0.028)	0.399 (0.033)	-2.305	.033	.046	-0.52
Fz	0.371 (0.025)	0.402 (0.038)	-3.957	.001	.005	-0.88
Fp1	0.381 (0.036)	0.418 (0.034)	-4.663	<.001	.003	-1.04
F4	0.375 (0.032)	0.397 (0.025)	-2.671	.015	.026	-0.60
PO8	0.385 (0.021)	0.407 (0.028)	-3.460	.003	.006	-0.77
O2	0.381 (0.032)	0.400 (0.027)	-2.346	.030	.046	-0.52
PO7	0.392 (0.031)	0.414 (0.026)	-2.877	.010	.021	-0.64
Fp2	0.391 (0.039)	0.419 (0.029)	-3.734	.001	.005	-0.83
F3	0.372 (0.028)	0.403 (0.030)	-3.456	.003	.006	-0.77
FC5	0.388 (0.029)	0.407 (0.030)	-2.799	.011	.022	-0.63
AF8	0.399 (0.041)	0.418 (0.028)	-2.253	.036	.047	-0.50

*p<.05, **p<.01, ***p<.001 (after FDR correction).

**Figure 4:**
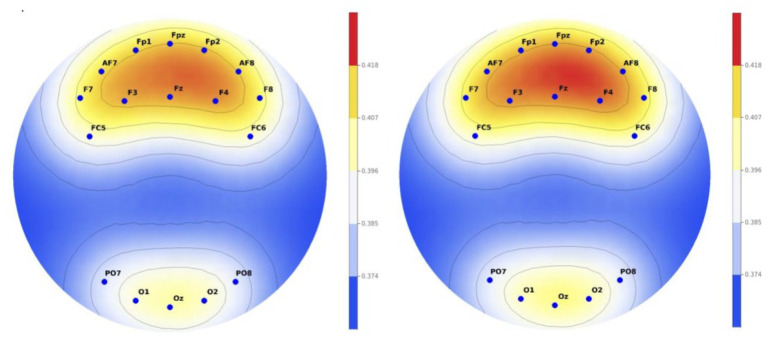
Comparison of EEG activity between two groups in the beta wave (left, normal music video; right, VR music video).

As a result of the analysis, there was a difference in beta wave between the group who watched normal music video and the group who watched the VR music video. It was confirmed that the group who watched the VR music video had a higher beta wave than the group who watched the normal music video. All 13 electrodes demonstrated medium to large effect sizes (Cohen’s d = 0.50-1.04 in absolute values), with the largest effects in prefrontal regions (Fp1: d = -1.04, Fpz: d = -0.89, Fz: d = -0.88), indicating substantial increases in cognitive arousal and concentration during VR viewing across widespread cortical regions (**Figure 4**).

### 3.3 EEG analysis results between two groups in theta/beta ratio

As a result of analyzing the difference in theta/beta ratio between the two groups, there was a significant difference between general K-pop content (NX group) and VR K-pop content (VR group) in specific brain regions. After FDR correction (q < 0.05), 8 out of 17 electrodes showed statistical significance (one more than the original 7 uncorrected results, as F3 became significant after correction). The specific brain regions include a total of 8 regions: AF7, Fpz, Fz, Fp1, PO8, PO7, Fp2, and F3, as shown in **Table 3**.

**Table 3:** EEG analysis results between two groups in theta/beta ratio.

Electrode	Normal (NX) M (SD)	VR M (SD)	t-statistic	p-value	p-FDR	Cohen's d
AF7	1.27 (.43)	1.68 (.64)	2.983**	.008	.023	0.67
Fpz	1.15 (.35)	1.48 (.53)	3.471**	.003	.015	0.78
Fz	1.06 (.22)	1.27 (.36)	2.830**	.011	.023	0.63
Fp1	1.16 (.38)	1.61 (.63)	3.910***	.001	.015	0.87
PO8	1.13 (.21)	1.35 (.30)	2.866**	.010	.023	0.64
PO7	1.22 (.34)	1.49 (.47)	3.058**	.007	.023	0.68
Fp2	1.28 (.48)	1.61 (.50)	3.547**	.002	.015	0.79
F3	1.14 (.27)	1.39 (.41)	2.916**	.009	.023	0.65

*p<.05, **p<.01, ***p<.001 (after FDR correction).

**Figure 5:**
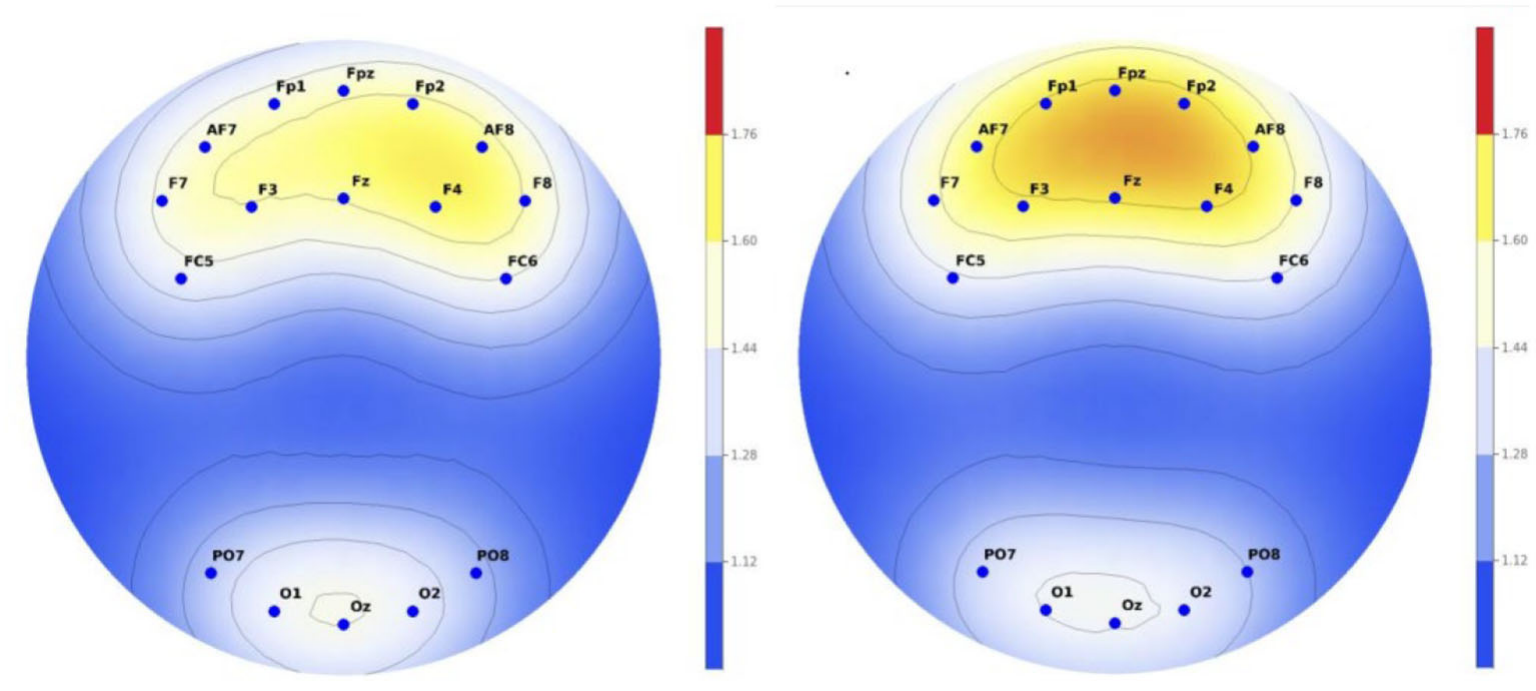
Comparison of EEG activity between two groups in theta/beta ratio (left, normal content; right, VR content).

As a result of the analysis, there was a difference in theta/beta ratio between the group who watched the normal music video and the group who watched the VR music video. It was confirmed that the group who watched the VR music video had a higher theta/beta ratio than the group who watched the normal music video. All 8 significant electrodes demonstrated medium to large effect sizes (Cohen’s d = 0.63-0.87), with the largest effects in prefrontal regions (Fp1: d = 0.87, Fp2: d = 0.79, Fpz: d = 0.78), indicating enhanced attentional engagement and flow states during VR viewing (**Figure 5**).

## 4 Discussion

In 2012, Psy’s Gangnam Style showed the potential of K-pop’s entry into the international market, and BTS became a global success, topping the Billboard charts for three consecutive years from 2018 to 2019. The global success of K-pop has brought enormous economic returns and cultural ripples to Korea (Lee and Jang, 2019). However, the success of K-pop in the international music market has a short history, and there are not many singers and groups that have achieved such success, so the continuity of K-pop’s success in the future is uncertain.

To overcome these limitations, K-pop labels establish a creative system by setting the main theme of the album and discovering and nurturing artists suitable for the set album to create more trendy songs. There are also many collaborations between K-pop artists and world-class musicians. Among these efforts the strategy of producing K-pop music videos incorporating virtual reality technology, one of the metaverse contents, is expected to have an important impact on the continued success of K-pop in the future (Kang et al., 2021).

Therefore, this study was conducted with EEG analysis to verify the influence of VR K-pop music videos as media based on the Media Richness Theory. Importantly, rigorous statistical methods were employed, including False Discovery Rate (FDR) correction for multiple comparisons to control for false positive findings across the 31 statistical tests conducted. This methodological rigor ensures that the reported findings represent genuine neurophysiological effects rather than statistical artifacts, addressing a critical gap in the neuroscience literature where multiple comparison corrections are often overlooked.

### 4.1 Alpha wave findings: cognitive familiarity and processing load

First, it was found that there was a difference in alpha waves between the two groups who experienced Aespa’s normal music video and VR music video. Specifically, the group that experienced the normal music video had higher alpha waves in 11 areas, including AF7, Fpz, F7, Fz, Fp1, F4, PO7, Fp2, F3, F8, and AF8, than the group who experienced the VR music video. After FDR correction, all 11 electrodes maintained statistical significance (q <.05), with effect sizes ranging from medium to very large (Cohen’s d = 0.54-1.51). The largest effects were observed in prefrontal regions (Fpz: d = 1.51, Fp1: d = 1.25, Fp2: d = 1.06), indicating particularly strong differential processing.

When a participant experiences familiar content that does not require the process of interpreting new content or information, alpha waves increase in areas located in the frontal lobe of the brain. This indicates light sleep and a stable state of mind (Kang et al., 2021). Based on these prior studies, the participants are in a more comfortable state when experiencing the normal music video. Through these results, it was found that the normal music video was more familiar to the participants than the VR music video and showed a stable psychological state, and that the cognitive process such as the interpretation of the lyrics of the song did not actively occur.

Relatively low alpha waves show that active cognitive phenomena appeared while watching unfamiliar VR music videos than normal music videos. It can be said that the participants perceived the virtual reality music video as a new form of unfamiliar content. This pattern aligns with the well-established phenomenon of “alpha blocking, “ whereby alpha waves decrease when the brain actively engages with novel or demanding stimuli (Red’ka and Mayorov, 2015; Rothschild and Hyun, 1990). The very large effect sizes observed (particularly for Fpz and Fp1, with d > 1.2) underscore that these are not merely statistically significant findings but represent substantial, practically meaningful differences in how the brain processes these two types of media. From a Media Richness Theory perspective (Daft and Lengel, 1986), the reduced alpha activity during VR viewing indicates that VR content, with its 360-degree spatial information and enhanced depth cues, demands greater attentional resources and cognitive engagement than traditional 2D viewing.

### 4.2 Beta wave findings: enhanced arousal and concentration in VR

Second, there was a difference in beta waves between the two groups who experienced Aespa’s normal music video and VR music video. Specifically, the group that experienced the VR music video had higher beta waves in 13 areas, including AF7, Fpz, F7, Fz, Fp1, F4, PO8, O2, PO7, Fp2, F3, FC5, and AF8 than the group who experienced the normal video. All 13 electrodes maintained significance after FDR correction, with medium to large effect sizes (Cohen’s d = 0.50-1.04 in absolute values). The largest effects were in prefrontal regions (Fp1: d = 1.04, Fpz: d = 0.89, Fz: d = 0.88).

During mental activities such as mental arithmetic, concentration, or tension, beta waves appear extensively in the cerebral cortex and indicate a stressful mental state (Moon and Choi, 2016). The relatively high beta wave when the subject experienced VR music video means high thinking activity, tension, and concentration. These results indicate that the participant is watching the VR music video in an awake state despite recognizing that it is virtual reality and is concentrating on virtual reality to which visual stimulation is applied.

The substantially elevated beta activity during VR viewing is particularly noteworthy given that participants were simply viewing content (not performing a demanding cognitive task), yet showed brain activity patterns consistent with active cognitive processing. The broad spatial distribution of beta increases across frontal, parietal, and occipital cortices suggests that VR engagement involves multiple cognitive systems: executive control (frontal), spatial processing (parietal), and visual processing (occipital). This multi-system activation distinguishes VR from traditional media viewing, which primarily activates visual processing regions.

Several theme parks in Germany and the United States offer roller coasters combined with virtual reality where people can see aliens from space wearing head-mounted displays. This is causing great satisfaction by providing new experiences and services to customers (Chung, 2017). Therefore, K-pop music videos applied with virtual reality technology can stimulate customers’ desire to purchase metaverse content. This has the potential to replace the existing K-pop music video content and can be a new powerful marketing strategy.

### 4.3 Theta/beta ratio findings: attentional engagement and flow states

Third, there was a difference in theta/beta ratio between the two groups who experienced Aespa’s normal music video and VR music video. The group who watched the VR music video had higher theta/beta ratio in specific brain areas than the group who watched the regular music video. After FDR correction, 8 electrodes showed significant differences (q <.05), with medium to large effect sizes (Cohen’s d = 0.63-0.87). Note that F3 became significant after FDR correction despite not reaching significance in the original uncorrected analysis, demonstrating the value of appropriate statistical correction.

The theta/beta ratio is an indicator of the participants’ attention span, flow state, and concentration (Friel, 2017). The high theta/beta ratio when experiencing the VR music video means that the participants’ attention span, flow state and concentration was higher than when they experienced the music video. Also, the theta/beta ratio is higher during uncontrolled thinking compared to controlled thinking (Angelidis et al., 2018). The participants experience the content with free and expanded thinking in a virtual space rather than reality, and this leads to concentration and flow state.

The direction of this finding requires nuanced interpretation. While elevated theta/beta ratios are associated with attentional difficulties in clinical populations (e.g., ADHD), in the context of healthy young adults viewing engaging entertainment content, the elevated theta/beta ratio during VR viewing likely reflects flow states characterized by immersive engagement and spontaneous, exploratory cognition. Unlike the focused, controlled attention required for demanding cognitive tasks (which would show lower theta/beta ratios), VR music video viewing may induce a more open, receptive attentional state that allows for deep experiential immersion. The neural signature we observed—elevated theta/beta ratio alongside elevated beta activity—suggests a unique cognitive state: heightened arousal (high beta) combined with immersive, exploratory attention (elevated theta/beta ratio). This interpretation aligns with phenomenological accounts of VR experiences, where users report feeling “transported” into virtual environments and experiencing a sense of presence that differs qualitatively from traditional media viewing.

In this regard, Japanese futurist Morinosuke Kawaguchi (2017) said that with the development of science and technology, content fused with virtual reality can create the ideal world and religious beliefs desired by the public. In addition, these virtual reality contents support the results of this study as they are highly likely to be spotlighted by modern people with new science and technology-friendly tendencies. It is necessary to develop devices and software that can meet users’ expectations for virtual reality, and this can lead to increased consumers’ interest in VR content.

### 4.4 Integration with media richness theory

Summarizing these results, in the case of VR Next Level music video, the amount of information transmitted to the brain as expanded visual and auditory information increased, and it produced effects such as high concentration, immersion, and arousal in the subject. The convergent evidence across multiple EEG measures demonstrates that VR K-pop music videos: (1) convey more information (reduced alpha, indicating active processing vs. passive viewing), (2) induce greater cognitive engagement (elevated beta across widespread cortical regions), and (3) create immersive attentional states (elevated theta/beta ratios in frontal regions). These neural differences provide robust evidence that the VR Next Level music video is playing the role of a rich media that forms high interactivity and induces a deep sense of immersion for consumers, according to the media richness theory (Daft and Lengel, 1986).

From a Media Richness Theory perspective, the VR condition can be considered a richer medium because it provides more simultaneous cues (e.g., 360° spatial information, depth cues, and enhanced perceptual presence) than conventional 2D viewing. In line with this framework, reduced alpha power during VR viewing suggests increased cognitive processing demands, whereas elevated beta power and altered theta/beta ratios indicate heightened arousal and immersive attentional engagement, respectively.

Importantly, these findings survived rigorous FDR statistical corrections and showed medium to large effect sizes across all three EEG measures, demonstrating that VR content is not simply “normal video in a headset” but represents a qualitatively different media experience that engages the brain more intensively across multiple cognitive dimensions.

## 5 Conclusions

Among metaverse contents, virtual reality is being actively produced in the K-pop market. An EEG analysis was performed on 20 college students who experienced Aespa’s Next Level VR music video and the normal music video. This study represents a methodological advance in EEG research on media psychology by applying rigorous multiple comparison corrections (FDR method) to ensure the validity of findings. The convergent evidence from alpha suppression, beta enhancement, and elevated theta/beta ratios provides robust support for Media Richness Theory’s applicability to VR content.

First, the group that experienced Aespa’s normal music video showed higher alpha waves than the group that experienced VR music video in 11 brain regions. After FDR correction (q <.05), all 11 electrodes maintained statistical significance with medium to very large effect sizes (Cohen’s d = 0.54-1.51), with the largest effects in prefrontal regions. These results mean that the participants who experienced VR music video perceived it as a new type of content that is unfamiliar compared to normal music video, and went through a new cognitive process.

Second, it was found that the group that experienced Aespa’s VR Next Level music video showed higher beta waves than the group that experienced the normal music video in 13 brain regions. All 13 electrodes remained significant after FDR correction with medium to large effect sizes (Cohen’s d = 0.50-1.04 in absolute values), indicating heightened concentration, arousal, and cognitive engagement during VR viewing across widespread cortical regions.

Third, the group that experienced Aespa’s VR Next Level music video had a higher theta/beta ratio than the group that experienced normal music video. After FDR correction, 8 electrodes showed significant differences with medium to large effect sizes (Cohen’s d = 0.63-0.87), suggesting immersive attentional states and flow experiences during VR viewing.

VR music videos provide richer visual and auditory information through the medium of virtual reality. This induces high interactivity for virtual reality experiencers, provides positive influences such as concentration and immersion, and proves that it is a rich media content presented in the Media Richness Theory. To provide greater satisfaction to customers, it is necessary to present a virtual reality environment that can utilize the five senses including sight and hearing. Therefore, it is essential to develop devices and software that can meet people’s high expectations for virtual reality content. To implement this and succeed, various government policy support is required, and investment and interest from private enterprises are important factors.

## 6 Limitations and future directions

This study has several methodological limitations that should be acknowledged. First, the within-subject design employed a fixed order (normal video followed by VR video for all participants). However, because the exposure order was fixed for all participants (NX→VR), the observed differences may have been partially influenced by order effects such as novelty, habituation/learning, or fatigue, despite the inclusion of the 180-s recovery interval. Future research should implement a counterbalanced sequence (NX→VR vs VR→NX) and formally test order effects by including sequence/order as a factor (e.g., mixed-design ANOVA or linear mixed models).

Second, limiting the participants of this study to male college students limits the generalizability of the outcomes. The exclusion of female participants, while necessary due to medication use that could confound EEG measures, limits our understanding of potential sex differences in VR engagement. Future research should therefore include a more diverse age group and both sexes to assess whether the observed VR effects generalize across demographic groups.

Third, only the Next Level music video of Aespa was used in this study. While this controls for content-specific confounds, it limits generalizability across different K-pop artists, genres, and visual styles. Additional, diverse VR K-pop music videos should be included in a follow-up study to evaluate brain-wave changes according to the type, mood, and genre of VR K-pop music videos.

Fourth, the VR content source used in this study was selected from available YouTube VR content, which may not represent optimal VR production standards. Purpose-designed VR content with professional production values and standardized technical specifications might show even stronger or more consistent effects.

Fifth, while our sample size (n = 20) was adequate for detecting medium to large effects in primary analyses and aligns with established guidelines for EEG studies (Larson and Carbine, 2017), it provides limited statistical power for exploratory subgroup analyses. Future studies with larger samples (n > 50) could investigate individual differences that might moderate VR engagement effects.

EEG band-power and ratio metrics exhibit notable inter-individual variability and are sensitive to contextual factors; therefore, the present findings should be interpreted as group-level correlates of media engagement in a non-clinical setting rather than as diagnostic or universally generalizable neural signatures. Participants’ cultural background and lived experience (e.g., familiarity with K-pop and prior exposure to VR) may modulate engagement-related EEG responses, which may limit replicability and generalizability. Future research should quantify media familiarity and include more diverse cultural contexts and viewing habits.

Despite these limitations, the rigorous statistical approach employed—including FDR correction for multiple comparisons and calculation of effect sizes—ensures that the reported findings represent genuine neurophysiological effects rather than statistical artifacts. The consistency of medium to large effect sizes across all three EEG measures and across multiple brain regions provides confidence in the robustness and replicability of these results.

## Data Availability

The original contributions presented in the study are included in the article/supplementary material. Further inquiries can be directed to the corresponding author.
